# Enrichment of KIR+CD57+ highly cytotoxic NK cells in sentinel lymph nodes of melanoma patients

**DOI:** 10.1186/1479-5876-12-S1-P10

**Published:** 2014-05-06

**Authors:** Talib Hassan Ali, Simona Pisanti, Elena Ciaglia, Roberta Mortarini, Andrea Anichini, Mario Santinami, Elio Gulletta, Caterina Ietto, Mario Galgani, Cinzia Garofalo, Giuseppe Matarese, Maurizio Bifulco, Soldano Ferrone, Francesco Colucci, Alessandro Moretta, Klas Kärre, Ennio Carbone

**Affiliations:** 1 Department of Experimental and Clinical Medicine “G. Salvatore”, University of Catanzaro ‘Magna Graecia’, Catanzaro, Italy; 2 Department of Microbiology, College of Medicine, University of Thi-Qar, Nasseriah, Iraq; 3 Department of Pharmaceutical and Biomedical Sciences, University of Salerno, Salerno, Italy; 4 Human Tumors Immunobiology Unit, Dept. of Experimental Oncology and Molecular Medicine (R.M. and A.A.), and Melanoma and Sarcoma Unit, Dept. of Surgery (M.S.), Fondazione IRCCS Istituto Nazionale dei Tumori, Milan, Italy; 5 Istituto di Endocrinologia e Oncologia Sperimentale, Consiglio Nazionale delle Ricerche (IEOS-CNR), Naples, Italy; 6 Department of Medicine, University of Salerno, Salerno, Italy; 7 Department of Surgery, Immunology and Pathology, University of Pittsburgh Cancer Institute, Pittsburgh, Pennsylvania, USA; 8 Department of Obstetrics and Gynaecology, University of Cambridge Clinical School, Cambridge, UK; 9 Lab. of Molecular Immunology, Department of Experimental Medicine, University of Genova, Genova, Italy; 10 Department of Microbiology Tumor and Cell Biology, Karolinska Institute, Stockholm, Sweden

## Background

NK cells contribute to melanoma cell recognition and anti-tumor immunity, which is traditionally analyzed using human peripheral blood NK cells. An important checkpoint in the progression of malignant melanoma is the metastasis to lymph nodes.

## Materials and methods

To investigate the role of lymph node NK cells in disease progression, we analyzed frequency, phenotype and functions of NK cells purified from either tumor infiltrated lymph nodes or tumor-free ipsilateral lymph nodes of the same patients. Lymph node NK cells were compared to peripheral blood NK cells from either melanoma patients or healthy donors.

## Results

The data showed an expansion of CD56^dim^CD57+CD69+CCR7+KIR+ NK cells in tumor infiltrated lymph nodes. This phenotype corresponds to a recently described fully mature and highly cytotoxic NK cell population, and indeed we found that these lymph node NK cells displayed robust anti-tumor activity against autologous melanoma cells. The NK cells trafficking from periphery to the tumor draining lymph nodes have been investigated and the chemokines pattern identified. Moreover, the presence of a high proportion of KIR+CD57+CD56^dim^ in the infiltrated lymph nodes was associated with an improved patients’ survival.

## Conclusions

Our data suggest that NK cells from tumor infiltrated lymph nodes are attractive candidates to improve current NK cell-based immunotherapy of melanoma.

